# Comparison of the Mitophagy and Apoptosis Related Gene Expressions in Waste Embryo Culture Medium of Female Infertility Types

**DOI:** 10.3390/life14111507

**Published:** 2024-11-19

**Authors:** Duygu Kütük, Çağrı Öner, Murat Başar, Berkay Akcay, İbrahim Orçun Olcay, Ertuğrul Çolak, Belgin Selam, Mehmet Cincik

**Affiliations:** 1 Department of Histology and Embryology, Medical Faculty, Maltepe University, 34858 İstanbul, Turkey; 2 IVF Laboratory, Bahçeci Umut Assisted Reproduction Center, 34662 İstanbul, Turkey; 3 Department of Medical Biology, Medical Faculty, Kırklareli University, 39100 Kırklareli, Turkey; cagrioner@klu.edu.tr; 4 Department of Obstetrics, Gynecology & Reproductive Sciences, Medical Faculty, Yale University, New Haven, CT 06520, USA; 5 Department of Biostatistics, Medical Faculty, Eskişehir Osmangazi University, 26040 Eskişehir, Turkey; 6 Department of Obstetrics and Gynecology, School of Medicine, Acibadem Mehmet Ali Aydinlar University, 34752 İstanbul, Turkey

**Keywords:** apoptosis, mitophagy, humanin, infertility, waste embryo culture medium

## Abstract

Mitochondria is an important organelle for the oocyte-to-embryo transition in the early embryonic development period. The oocyte uses mitochondria functionally and its mitochondrial DNA (mtDNA) content as the main energy source in the embryo development at the preimplantation stage. The aim of this study is to compare mitophagic, apoptotic and humanin gene expressions from the culture medium fluid in which embryos are developed and monitored among normoresponder (NOR), polycystic ovary syndrome (PCOS), young and older patients with poor ovarian reserve (POR). The study groups consisted of infertile patients who applied to the Bahçeci Umut IVF Center as NOR (Control), PCOS, POR-Advanced (POR-A) and POR-Young (POR-Y). After the isolation of total RNA from the collected samples, MFN1, MFN2, PINK1, PARKIN, SMN1, SMN2, p53 and Humanin gene expressions were determined by Real Time-PCR. The average age of only the POR-A was determined to be higher than the NOR (*p* < 0.001). The MFN1, SMN2 (*p* < 0.05), Humanin and p53 gene expressions (*p* < 0.001) increased, while PINK1 gene expression decreased (*p* < 0.05), in the POR-Y compared to the NOR. A decrease in MFN2, PARKIN (*p* < 0.05) and PINK1 gene expressions was determined in the PCOS compared to the NOR (*p* < 0.001). Furthermore, a decrease was observed in MFN2, PINK1 (*p* < 0.001) and Humanin gene expressions compared to the NOR (*p* < 0.05). The current data are the first in the literature determining the apoptotic and mitophagic status of the oocyte. The current results prove that waste embryo culture fluid may provide a non-invasive profile for important cellular parameters such as mitochondrial dysfunction in female infertility. The evaluation of significant cellular parameters can be performed much earlier without any intervention into the embryo.

## 1. Introduction

The World Health Organization (WHO) defines infertility as a clinical inability to achieve pregnancy after regular unprotected sexual intercourse for 12 months or longer [1]. Pre-implantation embryos may generally stall at various developmental stages due to reasons such as the patient’s age, ovarian stimulation protocols, inadequate in vitro culture conditions, the oocyte not reaching sufficient maturity, and paternal factors [2,3,4]. The embryo culture medium used in IVF during the first 3 days after fertilization can affect the size of the fetus, the birth weight, and even the postnatal weight at 2 years of age [4]. However, less is known about the effect of culture medium on embryonic growth and morphological development during the first trimester of pregnancy. This period is an important period in which rapid cell division, proliferation and differentiation occur for organogenesis [5]. In vitro embryo culture can induce epigenetic changes in different species, highlighting the need for caution in human IVF [6].

Energy metabolism is important in the development and maturation of oocytes. Mitochondria play a critical role in energy metabolism as well as cellular adaptation, organismal health, and longevity [7,8]. Although cumulus cells provide energy to oocytes via ATP, decreased mitochondrial function and reduced energy production capacity may cause oocyte aging. This may affect cell cycle regulation, spindle formation during mitosis, chromosome segregation, fertilization, embryo development, and implantation [2,9,10,11,12]. The quality of gametes, embryos, and the maternal environment for embryo implantation are critical parameters in the ability to achieve pregnancy or live birth. One of the proposed mechanisms responsible for ovarian aging is the accumulation of damage caused by reactive oxygen species (ROS), which is associated with mitochondrial dysfunction [13]. Mitochondrial dysfunction can lead to the failure of multiple cell organelles, apoptosis, and cellular senescence [14]. 

Apoptosis is one of the vital processes required to verify cell homeostasis, maintaining the balance between cell death and survival [15]. Mitochondria, Bcl-2 and caspase family members, cytochrome C, and p53 are key activators of the intrinsic pathway of apoptosis, which involves pro- and anti-apoptotic proteins [16]. p53 is at the center of multiple signaling pathways triggered by a range of cellular stresses, including DNA damage by exogenous mutagens, oncogene activation, telomere erosion, and hypoxia. All of these factors affect the abundance, subcellular localization, post-translational modification, and/or interaction of p53. p53 acts as a transcription factor in the nucleus or an inducer of apoptosis in the cytoplasm through different post-translational modifications [17]. Inappropriately activated p53-dependent apoptosis causes developmental abnormalities during the embryonic and postnatal period [18]. p53 activities increase fidelity and homeostasis in somatic tissue-specific stem and progenitor cells with transition from the germline to somatic tissues [19].

Survival motor neuron (SMN) is a ubiquitous protein that functions inside and outside the nervous system and has multiple cellular roles in transcription, translation, and proteostatic mechanisms. Due to a complex and highly repetitive DNA sequence, the human genome contains an inverted duplication in the SMN region of chromosome 5, producing a nearly identical duplicate gene, SMN2. Increased SMN2 copy numbers can partially compensate for the SMN1 mutation [20]. Further involvement of the SMN protein at the molecular level can be inferred through SMN interactions with known apoptotic proteins. One SMN interactor involved in apoptosis is p53, the pro-apoptotic protein that sequentially regulates the Bcl-2 family of proteins. p53 is a transcription factor involved in cell stress. It can both induce apoptosis (nuclear p53) and suppress autophagy (cytosolic p53). This is partially dependent on the translocation of p53 to external and internal (matrix) mitochondrial locations, the release of cytochrome c and the subsequent activation of caspases 3/7 [21]. Mitochondrial dysfunction or DNA mutation in animal and cell models can occur due to endogenous stimuli such as hypoxia, Ca^2+^ overload, or oxidative stress [22]. 

Mitophagy is the main pathway for the degradation of dysfunctional or unnecessary mitochondria in cells. The PINK1/PARKIN pathway is the most extensively characterized mechanism affecting mitochondrial quality control in cells [23]. PINK1 (PTEN-induced kinase 1) and PARKIN (E3 ubiquitin protein ligase), two autosomal recessive PD-associated genes, have been linked to mitochondrial quality control. In healthy mitochondria, PINK1 is transferred to the matrix, where it is released back into the cytosol after degradation by proteases [24,25]. 

The decrease in mitochondrial membrane potential is the most important indicator of mutated mitochondria. As a result of the mutation, the PINK-1 protein begins to accumulate on the outer membrane of the mitochondria. This accumulation enables the PARKIN to selectively bind to the mutated mitochondria. This binding causes the ubiquitination of two proteins located on the outer membrane of the mitochondria. The fusion of mutated mitochondria with intact mitochondria is blocked by the ubiquitination of MFN1 and MFN2, which are PARKIN substrates, and apoptosis of the cell is prevented [26]. Mitochondrial fusion in mammals is controlled by MFN1 and MFN2, proteins located in the outer mitochondrial membrane. When MFN1 expression is reduced, mitochondrial fusion can be rescued by MFN2 overexpression and vice versa [27]. 

Humanin (HN) is a mitochondrial-derived peptide produced in response to cellular stress [28]. HN is encoded by a 75-bp region in an open reading frame within the mitochondrial 16S ribosomal RNA [29]. HN has antiapoptotic and neuroprotective effects [30,31]. The HN peptide contains 21 amino acids when its mRNA is translated in the mitochondria and 24 amino acids when its peptide is transported to the cytoplasm [32]. 

HN regulates many pathways, including apoptosis, in various diseases such as growth hormone-, cancer-, and brain-related diseases. Several recent studies in rats revealed that HN translation occurs in mitochondria, which shows definitive antiapoptotic activities. Since its discovery in 2001, the role of HN has been demonstrated in many biological processes, including oxidative stress and anti-apoptosis [33]. Over the past few years, HN has become an impressive therapeutic agent due to its cytoprotective, reactive oxygen species and antiapoptotic effects in various cell types, including neuronal, male germ cells, and cardiac cells. HN deficiency contributes to certain aging processes, including cellular senescence, chronic inflammation, and cognitive decline [34]. HN increases long-lived protein degradation through the stimulation of both basal and inducible autophagy. HN plays an important role in regulating the cellular response to oxidative stress and apoptosis in the ovaries and testes through the modulation of various signaling pathways, especially when the body is in an abnormal state [15]. 

The non-invasive evaluation of oocytes and embryos with high developmental potential in IVF-ET procedures is important to reduce embryo transfer failure rates and improve pregnancy outcomes [35]. The existing approaches to gauging the potential for embryo implantation primarily hinge on morphological assessment. However, this method does not consistently provide accurate predictions of successful implantation [36]. In order to make a comprehensive assessment of embryo quality, it is possible to make the selection more accurate and consistent by analyzing embryo metabolomics as well as standard morphokinetic results [37,38]. 

Mitochondria play a central and multifaceted role in the mammalian egg and early embryo, contributing to many different aspects of early development [39]. Various methods have been used to measure oocyte mitochondria, all of which have significant limitations. However, the few studies that have examined mitochondrial number in relation to oocyte developmental competence suggest that higher-quality oocytes contain more mitochondria [40]. Several studies have already established correlations between embryonic viability and carbohydrate, pyruvate, and amino acid metabolism during embryonic development. Indeed, the metabolic performance of embryos post-compaction has emerged as a noteworthy biomarker indicative of superior-quality blastocysts [36]. 

Previous studies have shown that nuclear magnetic resonance spectroscopy was used to assess glucose levels in follicular fluid of PCOS patients, and that there was a positive correlation between low glucose uptake by germ cells and the embryonic development rate [35].

In another study, after single blastocyst transfer on day 5, spent culture medium was subjected to metabolite analysis using nuclear magnetic resonance (NMR) spectroscopy. Combining ML models with metabolomic and embryological data has been reported to improve the prediction of embryo implantation potential, and can be used to achieve clinical benefits for patients in real time [41].

The autophagic removal of mitochondria appears to be one of the cellular mechanisms that prevent loss of muscle mass and quality by attenuating mitochondria-dependent apoptosis in a healthy cell [42]. The impact of mitochondrial dysfunction on embryo quality is investigated to predict the developmental competence and implantation potential of the embryo [43,44]. This relationship demonstrates the power of using metabolomics in IVF culture media for non-invasive embryo selection [45]. In this study, we investigated the impact of mitophagy-related genes and determined the gene expression profiles by using waste embryo culture medium between female infertility groups, which are NOR, PCOS, POR-Y and POR-A.

## 2. Materials and Methods

### 2.1. Sample Collection

Human waste embryo culture medium samples donated to research were obtained with informed consent from patients undergoing IVF at the Bahceci Health Group Umut IVF Laboratory, Istanbul. This study was approved by the Ethics Committee of the Maltepe University (2022/20-05 and 2023/19-22), and written informed consent was signed by each patient. Here, 200 waste embryo culture medium samples belonging to normoresponder (NOR), polycystic ovary syndrome (PCOS), young and advanced patients with low ovarian reserve (POR-Y and POR-A) were analyzed at Bahçeci Umut In Vitro Fertilization Center, with 50 patients in each group. Inclusion criteria for NOR patients (control group) were that they must have antral follicle count (AFC) 7-12, and anti-Mullerian hormone (AMH) levels 1,0-3,5 ng/mL. PCOS patients must have AFC > 12, and AMH levels >3.5 ng/mL. POR-Y patients must have AFC< 7, AMH levels > 1 ng/mL, and age < 35, and POR-A patients must have AFC < 7, AMH levels >1 ng/mL, and age > 35.

### 2.2. Ovarian Stimulation, Oocyte Retrieval, Intracytoplasmic Sperm Injection (ICSI) and Embryo Culture

Ovarian stimulation (OS) was started on day 2 of the menstrual period by employing the antagonist protocol. The dosage of gonadotropins was individualized based on the patients’ parameters. Patients received 250 µg of human chorionic gonadotropin (hCG; Ovitrelle, Serono) or 0.2 mg of triptorelin (Gonapeptyl, Ferring) for the final oocyte maturation when at least two follicles reached 18 mm in diameter, and transvaginal ultrasound-guided follicle aspiration was performed for oocyte retrieval after 35 h. The oocyte retrieval, denudation, and ICSI procedures were performed as previously described [38]. After microinjection, oocytes were cultured individually in a unique, pre-equilibrated culture dish. Single-step media—namely, single continuous culture complete (CSCM-C) with human serum albumin (Irvine Scientific, Santa Ana, CA, USA)—was used for the embryo cultures throughout the culture period in our study. All embryos were kept in benchtop incubators (MIRI, ESCO Medical, Singapore) and cultured until day 5 or 6 of embryo development. Waste embryo culture medium materials were transferred into a PCR tube and kept frozen at 20 °C until analysis.

### 2.3. Morphological Classification of Embryos

On day 3 of embryo development, a division stage morphological score was assigned based on a three-point grading system using characteristics such as cell number, fragmentation, symmetry, and shape. The morphological score was based on the expansion stage, the quality of the inner cell mass (ICM), and the quality of the trophectoderm (TE) at the blastocyst stage.

### 2.4. The Waste Embryo Culture Medium Collection

The culture medium of embryos was collected after finishing the culture. Single culture droplets had been overlaid with oil; thus, pipetting of the medium had to be performed to avoid taking the oil. Spent embryo media was collected with gel pipette tips. The waste embryo culture mediums were collected and were stored at −20 °C.

### 2.5. Total RNA Isolation and Real Time Polymerase Chain Reaction (RT-PCR)

Total RNA was obtained using an innuPREP Micro RNA Kit (AnalytikJena, Germany) according to the manufacturer’s instructions. Total RNAs were reverse-transcribed into cDNA (Nucleogene, Turkey). SYBR Green primer sets for the amplification of MFN1, MFN2, SMN1, SMN2, PINK1, PARKIN, Humanin, p53 and Glyseraldehide-3-phosphate dehydrogenase (GAPDH) were designed and supplied by Bmlabosis (Ankara, Turkey). The primer sequences are shown in Table 1. RT-PCR was performed in Roche Lightcycler96 (Vedbaek, Denmark). The ΔCT formula was used to determine gene expressions.

### 2.6. Statistical Analysis

The Kolmogorov–Smirnov test was used to determine if the continuous variables had a normal distribution. Using one-way variance analysis, comparisons between groups of normally distributed variables were assessed. The software program IBM SPSS Statistics 26.0 was used for all analyses. The acquired data were presented as the mean ± standard deviation.

## 3. Results

### 3.1. The Evaluation of Ages Between NOR, PKOS, POR-Y and POR-A Samples

**Figure 1:**
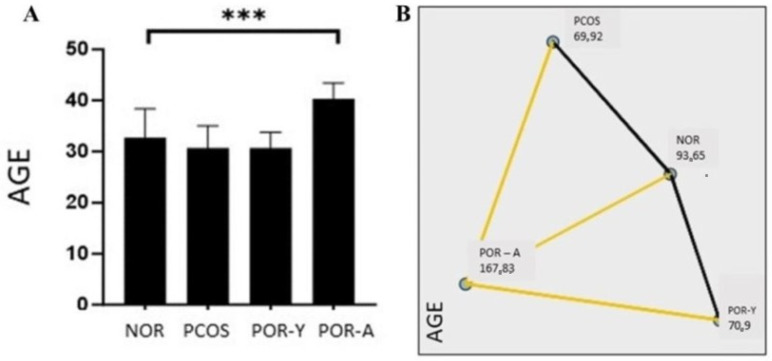
(**A**) Comparison of the ages among the samples from NOR, PCOS, POR-Y and POR-A groups. (**B**) Multiple comparison of ages of samples from NOR, PCOS, POR-Y and POR-A groups *** *p* < 0.001; NOR—normoresponder (Control) group; PCOS—polycystic ovary syndrome; POR-Y—poor responder young age patients; POR-A—poor responder advanced age patients).

A statistically significant increase was observed in POR-A (40.36 ± 3.082) compared to NOR (32.74 ± 5.609; *p* < 0.001, Figure 1). No statistically significant decrease was observed in the PCOS (30.68 ± 4.349) or POR-Y (30.70 ± 3.078) compared to NOR (*p* > 0.05, Figure 1A). The mean age in the POR-A (40.36 ± 3.082) group showed a significant increase compared to the PCOS (30.68 ± 4.349) and POR-Y (30.70 ± 3.078; *p* < 0.001, Figure 1B).

### 3.2. Determination and Comparisons of Gene Expression Profiles Between NOR, PKOS, POR-Y and POR-A Samples

**Figure 2:**
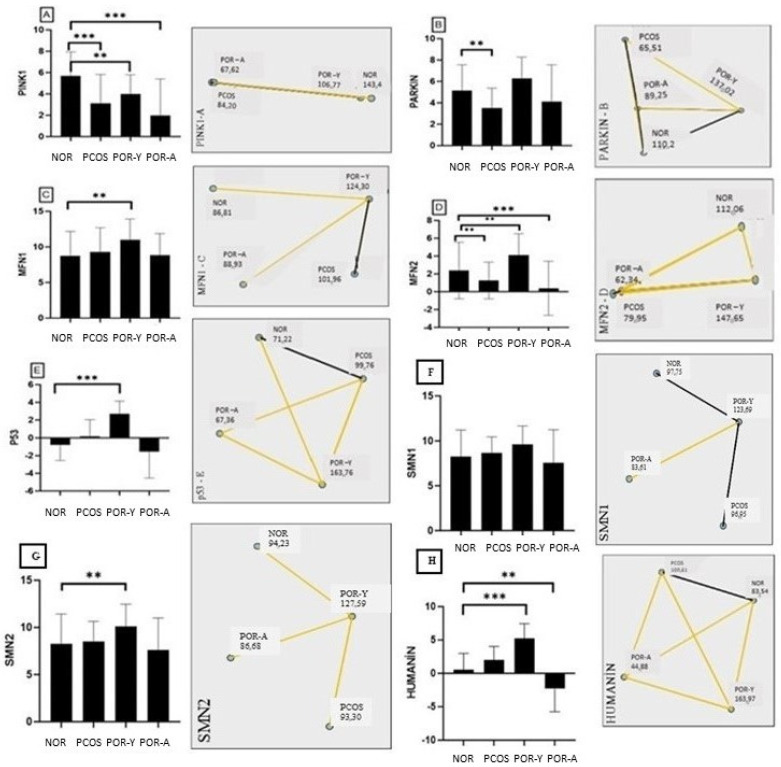
(**A**) Gene expression and multiple comparisons of PINK1 between NOR, PCOS, POR-Y and POR-A. (**B**) Gene expression and multiple comparisons PARKIN between NOR, PCOS, POR-Y and POR-A. (**C**) Gene expression and multiple comparisons MFN 1 between NOR, PCOS, POR-Y and POR-A. (**D**) Gene expression and multiple comparisons of MFN 2 between NOR, PCOS, POR-Y and POR-A. (**E**) Gene expression and multiple comparisons of p53 between NOR, PCOS, POR-Y and POR-A. (**F**) Gene expression and multiple comparisons of SMN1 between NOR, PCOS, POR-Y and POR-A. (**G**) Gene expression and multiple comparisons of SMN2 between NOR, PCOS, POR-Y and POR-A. (**H**) Gene expression and multiple comparisons of Humanin between NOR, PCOS, POR-Y and POR-A. (** *p* < 0.05 and *** *p* < 0.001; NOR—normoresponder (Control) group, PCOS—polycystic ovary syndrome, POR-Y—poor responder young age patients, POR-A—poor responder advanced age patients).

PINK 1 gene expression significantly decreased in the PCOS (3.11 ± 2.715), POR-A (1.98 ± 3.421; *p* < 0.001) and POR-Y (3.98 ± 1.811; *p* < 0.05) compared to the NOR (5.68 ± 2.253, Figure 2A) group. The PINK 1 gene expression in the POR-Y (3.98 ± 1.811) group significantly increased compared to the POR-A (1.98 ± 3.421; *p* < 0.05, Figure 2A).

PARKIN gene expression significantly decreased in the PCOS (3.51 ± 1.855) group compared to the NOR (5.14 ± 2.410); *p* < 0.05, Figure 2B). The PARKIN gene expression in the POR-Y (6.28 ± 1.982) group showed a significant increase compared to the POR-A (4.13 ± 3.420) and PCOS (3.51 ± 1.855; *p* < 0.001, Figure 2B) groups.

MFN 1 gene expression significantly increased in POR-Y (10.9853 ± 2.937) compared to NOR (8.73 ± 3.433; *p* < 0.05, Figure 2C). MFN 1 gene expression significantly increased in POR-Y (10.9853 ± 2.937) compared to POR-A (8.82 ± 3.034; *p* < 0.05, Figure 2C).

MFN 2 gene expression significantly decreased in POR-A (0.38 ± 3.037) compared to NOR (2.38 ± 3.177; *p* < 0.001) and PCOS (1.26 ± 2.064; *p* < 0.05). The mean MFN 2 gene expression in POR-Y (4.1049 ± 2.411) significantly increased compared to NOR (2.38 ± 3.177), POR-A (0.38 ± 3.037) and PCOS (1.26 ± 2.064; *p* < 0.05, Figure 2D).

p53 gene expression significantly increased in POR-Y (2.68 ± 1.453) compared to NOR (−0.78 ± 1.773; *p* < 0.001). The increase in the PCOS (0.17 ± 1.873) and POR-A (−1.55 ± 2.983) was not statistically significant (*p* > 0.05). p53 gene expression significantly decreased in PCOS (0.17 ± 1.873) compared to POR-Y (2.68 ± 1.453; *p* < 0.001) and significantly increased compared to POR-A (−1.55 ± 2.983; *p* < 0.05, Figure 2E).

SMN 1 gene expression in POR-Y (9.59 ± 2.055) significantly increased compared to POR-A (7.577 ± 3.670; *p* < 0.05, Figure 2F). 

SMN 2 gene expression significantly increased in POR-Y (10.11 ± 2.345) compared to NOR (8.27 ± 3.149; *p* < 0.05), PCOS (8.49 ± 2.151) and POR-A (7.62 ± 3.366; *p* < 0.05, Figure 2G).

HN gene expression significantly increased in POR-Y (5.19 ± 2.239; *p* < 0.001) compared to NOR (0.55 ± 2.437). A significant decrease was observed in POR-A (−2.32 ± 3.408; *p* < 0.05) compared to NOR (0.55 ± 2.437), PCOS group (2.02 ± 2.004) and POR-Y (5.19 ± 2.239). HN gene expression significantly decreased (*p* < 0.001) in the PCOS group (2.02 ± 2.004) compared to POR-Y (5.19 ± 2.239), and showed a significant increase compared to POR-A (−2.32 ± 3.408; *p* < 0.001, Figure 2H). 

To determine the strength and significance of the relationship between Spearman’s Rho distribution and gene expressions, both the coefficient value and the significance level were determined (Table 2). According to the correlation analysis of gene expression levels of NOR, PCOS, POR-Y and POR-A in Table 2, MFN1, MFN2, SMN1, SMN2, PINK1, PARKIN, Humanin and p53 genes showed positive correlations with each other (** r < 0.01). As the correlation level continued in the positive direction (+1), the expressions of the related genes increased in reverse or directly proportional ways (** r < 0.01).

## 4. Discussion

To our knowledge this is the first study in the literature determining the apoptotic and mitophagic status of the oocyte from waste embryo culture fluid and providing a non-invasive profile for important cellular parameters such as mitochondrial dysfunction in female infertility.

The decline in fertility over time results from ovarian ageing, which is characterized by the quantitative and qualitative alteration of the ovarian oocyte reserve [46]. When the average age of the patient groups was evaluated in the current study, only those in POR-A were found to be significantly older compared to the NOR group. The samples were collected ideally in terms of the age parameter. 

In addition to age and low ovarian reserve parameters, embryo waste culture fluid can be used non-invasively to estimate the adequacy of oocyte development in the etiology of biological maturation and to evaluate the potential of the embryo in the developmental process.

PINK1/Parkin are linked to mitochondrial quality control. The increased expression of PINK1, a marker of damaged mitochondria, is one of the indicators of the decrease in mitochondrial membrane potential. PINK1-PARKIN prevents apoptosis through mitophagy, improves mitochondrial biogenesis and contributes to embryo development [47].

Cumulus–oocyte complexes (COCs) can activate mitophagy in response to mitochondrial membrane depolarization [48]. Oocytes may respond to mitochondrial damage by increasing the number of mitochondria instead of eliminating them [49]. Determining a significant decrease in PINK1 gene expression level in PCOS, POR-Y and POR-A groups is an expected result. PINK1, which shows low gene expression levels in healthy cells, was predicted to be high in the POR-Y and POR-A groups where the apoptosis mechanism is active. Although mitophagy is a reversible mechanism, it leads to apoptosis during unexpected situations. POR-Y and POR-A groups escape mitophagy by tolerating this mechanism. The mitochondrial activity of embryos will protect the cell from apoptosis and necrosis as the number of mitochondrial DNA copies in the environment increases. Mitophagy is better tolerated in the POR-Y group due to the effect of the age factor. The number of oocytes increases in the PCOS group due to the increase in adipose tissue, but their quality is low. Although the mitophagic pathway is not assumed to be active here, low PINK1 expression supports these data. We determined a correlation between PARKIN and PINK1 (r < 0.01). Accordingly, a decrease in the expression of the PARKIN gene in the PCOS group is expected due to the low rate of mitophagy. The main reason for the difference between the PCOS and POR-Y groups is that the number of oocytes is low in the POR-Y group, that is, the mitophagic/apoptotic mechanism is active. Although PARKIN gene expression was higher in the POR-A group compared to PCOS, the reason for the lack of statistical difference may be the advanced age factor.

Mitofusins are recognized as decision-makers in the interaction between mitophagy and apoptosis. Mitofusins are reduced in disease states and their pathophysiology is improved by normalizing mitofusin levels, thus highlighting a broad therapeutic potential of these proteins [50]. MFN1 and MFN2 are associated with female fertility [51]. Oocyte-specific MFN1 deletion causes female infertility due to defective oocyte maturation and follicular development [52]. The deletion of MFN1 in oocytes leads to apoptosis following the disruption of gap junction connections between oocytes and granulosa cells, and results in accelerated follicular atresia [53]. 

The deletion of MFN2 in the oocyte leads to mitochondrial dysfunction and subfertility associated with follicle development and oocyte maturation. The absence of MFN2 in the oocyte increases apoptosis and the shortening of telomere length, leading to impaired oocyte quality and follicular depletion, a phenotype consistent with accelerated reproductive senescence [54]. 

MFN2 gene expression provides more information about mitophagy and apoptosis in all groups in our study. The increase in MFN1 gene expression only in the POR-Y group shows that the effects of mitophagy and apoptosis are less in this group than expected. The expressions of MFN2, PINK1 and PARKIN genes are also correlated with MFN1 gene expression. The decrease in MFN2 gene expression in PCOS and POR-A patients indicates that mitophagic pathways are suppressed. The suppression of POR-A indicates a different pathway from age-related apoptosis. The pathogenesis of POR-A occurs due to the disruption of this balance.

p53 plays a regulatory role in cell cycle arrest, immune regulation, diabetes, insulin resistance, aging and apoptosis [54]. The POR-Y group in the current study has higher p53 gene expression than NOR and other groups, which shows clearly that there is no effect of apoptosis or mitophagy on oocyte degeneration. The data show that the molecular infrastructure of the p53 gene and other genes do not have an effect in the young patient group with low ovarian reserve; on the contrary, this group is far from apoptosis. The distributions for p53 between PCOS and POR-Y and POR-Y and POR-A are higher than the POR-Y group, in which the apoptosis in the PCOS and POR-A distribution is collected. According to the information obtained, the increase in p53 gene expression of the POR-Y series indicates that the apoptotic process in the oocyte started at the follicular stage, and indicates both the size of the reserve and embryo development. Although the low numbers of oocytes obtained and the resulting embryos in the POR-Y group were explained by apoptotic/mitophagic pathways, the molecular parameters we examined demonstrated that neither of these two mechanisms are determinant in the development of the embryo in POR-Y.

The importance of SMN in humans was first recognized when deletions or mutations in the SMN1 gene caused Spinal Muscular Atrophy (SMA), a leading genetic disease. The SMN2 duplication of the SMN1 gene is almost universally present in patients. Therefore, SMN2 is the most promising therapeutic target. SMN reduction affects the testes and their development and function. The low expression of the SMN genes has recently been associated with testicular defects and male infertility. Both abnormally low and high SMN expression can result in pathological conditions [55]. 

SMN1 gene expressions do not show any statistical difference between all groups (*p* > 0.05). Although higher SMN1 gene expression was observed in the groups with low levels of apoptosis compared to the NOR group, it was observed that this situation was not at a level that would exceed the statistical threshold.

SMN may have a protective effect due to age, exposure to oxidative stress and decreases in oocyte quality, but higher SMN levels are needed. Higher SMN1 gene expression in groups with low levels of apoptosis was not significant compared to the NOR group in the current study. SMN1 gene expression profiles may be clarified after studying a larger number of samples to ensure the accuracy of the expression profile in the PCOS, POR-Y and POR-A groups. SMN2 gene expression increases in cases with high degeneration, due to excessive duplication of the relevant allele. High SMN2 gene expression in the POR-Y group supports this interpretation. Considering that SMN exhibits anti-apoptotic properties, the ability to obtain gene expression levels even in waste culture fluid suggests that it may have a protective effect on mitochondrial function by preventing apoptosis in POR-Y embryo development.

Apoptosis increases during folliculogenesis in individuals with PCOS, and the abnormal proliferation of granulosa cells (GCs) causes anovulation and infertility [56]. Oocyte morphology changes with decreased pregnancy and live birth rates in overweight or obese women [57]. Aging may affect cytoplasmic quality, nuclear chromosomal abnormalities, oocyte competence and mitochondria in particular [14]. The mtDNA contents in embryos at the early cleavage stage are lower among older women, compared to those in young women [46,49]. 

Paradoxically, the mtDNA content of blastocysts increases in older patients. Similarly, aneuploid blastocysts contain greater numbers of mtDNA copies than euploid blastocysts [58]. Mitochondrial proliferation may therefore be a compensatory mechanism for mitochondrial insufficiency [59]. 

HN is optimally expressed in the cytoplasm of oocytes in the early stage of postnatal development, and humanin expression decreases with increasing age [60]. HN concentration in follicular fluid is positively associated with ovarian reserve and clinical pregnancy rate [61]. Local ovarian HN expression in insulin-resistant PCOS patients is reduced compared to that in non-insulin-resistant PCOS patients [62]. 

According to our data, mtDNA gene HN showed different expression profiles depending on age. The high HN expression in POR-Y, which escapes apoptosis and has very low mitophagy, is normal because no destruction mechanism is active. The high mitochondria number in POR-Y, which may be age-related, indicates high humanin gene expression. We observed that HN gene expression decreased in POR-A due to the decrease in the number of mitochondria with aging.

Age-related accumulated mtDNA mutations, especially obtained from the POR-A, may be involved in ovarian failure. Advanced age can cause infertility and oocyte abnormalities in women. There is no negative effect of mitophagy and apoptosis mechanisms in PCOS samples. When mitophagy parameters are considered in this group, no significant decrease or increase is observed in the estimated mitochondria numbers, and humanin gene expression does not give a statistically significant result. 

The difference in HN expression between the groups in PCOS, POR-Y and POR-A indicates that oocyte maturation in the POR-Y samples is higher than in those with PCOS. HN gene expression, which indicates oocyte maturation, was determined to be higher in young PCOS samples compared to older samples with POR-A.

The basic molecular mechanisms underlying the conditions of patients with different indications presenting to IVF centers have not been fully elucidated. Understanding the molecular mechanisms is necessary for the diagnosis and treatment of diseases. Although a low number of oocytes and consequently the low number of developing embryos in the POR-Y may be explained by apoptotic/mitophagic pathways, the molecular parameters we examined showed that neither of these two mechanisms were determinant in embryo development for POR-Y. The molecular parameters examined demonstrated that the effects of age-related changes in the POR-A group caused a decrease in ovarian reserve. Examining the possible effect of the waste culture fluid of the non-developing embryos of POR-A patients on the mitophagy and apoptosis pathways may provide definitive information about the effect of the molecular substructure in this patient group.

The current study provides preliminary data showing that the relevant genes for SMA can be evaluated non-invasively before PGT in individuals who have a high potential for SMA in their children in the future. The present study reveals that waste embryo culture medium is a good candidate for the molecular diagnosis of female infertility subgroups. The use of waste embryo culture medium in the IVF treatment process can be considered as a possible additional option in terms of both short-term results and lower cost. We examined the effects of mitophagy and subsequent apoptosis, SMN gene-dependent degeneration and subsequent apoptosis, and humanin and mtDNA on embryo development at the molecular level. Genetic analysis can be performed to examine the various molecular pathways in embryo waste culture fluid non-invasively without any invasive biopsy material and any intervention into the embryo, providing advantages in terms of embryo development and follow-up.

## Figures and Tables

**Table 1:** Forward and reverse primer sequences used in RT-PCR.

Gene	Forward Sequence	Reverse Sequence
MFN1	5′-GTTACCGAGGAGGTGGCAAA-′3	5′-GGTCTGAAGCACTAAGGCGT-′3
MFN2	5′-TCTCCCGGCCAAACATCTTC-′3	5′-TCCATGTACTCGGGCTCTGA-′3
SMN1	5′-CACAGGCCAGAGCGATGATT-′3	5′-TGGAGCAGATTTGGGCTTGA-‘3
SMN2	5′-CACAGGCCAGAGCGATGATT-′3	5′-TGGAGCAGATTTGGGCTTGA-′3
PINK1	5′-TGGCTGGTGATCGCAGATTT-′3	5′-AGAGCGTTTCACACTCCAGG-′3
PARKIN	5′-CTGCCGGGAATGTAAAGAAGC-3′	5′-CCACAGTTCCAGCACCACTC-3′
HUMANIN	5′-CACTCCACCTTACTACCAG-′3	5′-ATAATTTTTCATCTTTCCC-′3
p53	5′-TGAAGCTCCCAGAATGCCAG-′3	5′-TGGTGTTGTTGGACAGTGCT-′3
GAPDH	5′-CGAGGGGGGAGCCAAAAGGG-′3	3′-GAAACTGCGACCCCGACCGT-′5

**Table 2:** Correlations for all gene groups (** r < 0.01).

Correlations For All Gene Groups										
			MFN1	MFN2	SMN1	SMN2	PINK1	PARKIN	HUMANIN	p53
Spearman’s Rho	MFN1	R	1.000	0.385 **	0.550 **	0.529 **	0.258 **	0.372 **	0.344 **	0.429 **
		Sig. (2-)	-	0.000	0.000	0.000	0.000	0.000	0.000	0.000
		N	200	200	200	200	200	200	200	200
	MFN2	R	0.385 **	1.000	0.536 **	0.489 **	0.549 **	0.599 **	0.606 **	0.668 **
		Sig. (2-)	0.000	-	0.000	0.000	0.000	0.000	0.000	0.000
		N	200	200	200	200	200	200	200	200
	SMN1	R	0.550 **	0.536 **	1.000	0.641 **	0.468 **	0.594 **	0.441 **	0.505 **
		Sig. (2-)	0.000	0.000	-	0.000	0.000	0.000	0.000	0.000
		N	200	200	200	200	200	200	200	200
	SMN2	R	0.529 **	0.489 **	0.641 **	1.000	0.405 **	0.498 **	0.411 **	0.511 **
		Sig. (2-)	0.000	0.000	0.000	-	0.000	0.000	0.000	0.000
		N	200	200	200	200	200	200	200	200
	PINK1	R	0.258 **	0.549 **	0.468 **	0.405 **	1.000	0.637 **	0.362 **	0.402 **
		Sig. (2-)	0.000	0.000	0.000	0.000	-	0.000	0.000	0.000
		N	200	200	200	200	200	200	200	200
	PARKIN	R	0.372 **	0.599 **	0.594 **	0.498 **	0.637 **	1.000	0.447 **	0.549 **
		Sig. (2-)	0.000	0.000	0.000	0.000	0.000	-	0.000	0.000
		N	200	200	200	200	200	200	200	200
	HUMANIN	R	0.344 **	0.606 **	0.441 **	0.411 **	0.362 **	0.447 **	1.000	0.724 **
		Sig. (2-)	0.000	0.000	0.000	0.000	0.000	0.000	-	0.000
		N	200	200	200	200	200	200	200	200
	p53	R	0.429 **	0.668 **	0.505 **	0.511 **	0.402 **	0.549 **	0.724 **	1.000
		Sig. (2-)	0.000	0.000	0.000	0.000	0.000	0.000	0.000	-
		N	200	200	200	200	200	200	200	200

## Data Availability

The authors declare that all data are embedded in the manuscript. The datasets generated during and/or analyzed during the current study are available from the corresponding author on reasonable request.
